# Quantification of the exposure and effects of road traffic noise in a dense Asian city: a comparison with western cities

**DOI:** 10.1186/s12940-015-0009-8

**Published:** 2015-03-07

**Authors:** Alan Lex Brown, Kin Che Lam, Irene van Kamp

**Affiliations:** Urban Research Program, Griffith School of Environment, Griffith University, Nathan, Brisbane, Australia; Department of Geography and Resource Management, The Chinese University of Hong Kong, Hong Kong, China; National Institute for Public Health and the Environment, Centre for Sustainability, Environment and Health, Bilthoven, The Netherlands

**Keywords:** Road traffic noise, Exposure, Exposure-response relationships, Annoyance, Sleep disturbance, Hong Kong, Megacities

## Abstract

**Background:**

Particularly in Asia, dense, traffic-intense, and usually high-rise cities are increasingly the norm. Is existing knowledge on exposure to road traffic noise, and on people’s response to such exposure, garnered primarily from western cities, equally applicable in these?

**Methods:**

Hong Kong has high population and traffic density and a high-rise building form. Road traffic noise exposure was estimated, and residents’ responses to traffic noise measured, for a sample of 10,077 dwellings. Noise level estimates were based on three-dimensional modelling. Best international survey practice measured self-reported annoyance and sleep-disturbance. Benchmark estimates of exposure, and of annoyance and self-reported sleep disturbance, are provided. We compare Hong Kong exposure with those of European cities, and the exposure-response relationship for annoyance in Hong Kong to those reported from elsewhere - based on the tolerance limits of previous syntheses. Exposure-response for self-reported sleep disturbance is also compared.

**Results:**

The distribution of exposures of dwellings in high-rise, high-density, Hong Kong is different from those reported from Europe, but not at the higher noise levels. The exposure-annoyance relationship for road traffic noise was from the same population of exposure-response relationships, being well within the tolerance limits, of studies used to generate the synthesized Miedema and Oudshoorn curves. The exposure-response curve for self-reported sleep disturbance was parallel to that of Miedema and Vos but slightly lower.

**Conclusions:**

The proportion of the Hong Kong population exposed to high levels (>70 dB) is similar to that found in Europe. However, a much higher proportion, compared to European cities, is exposed to L_den_ levels of 60–64 dB, and a much lower proportion to lower levels (<55 dB). There is no evidence that the exposure-response relationships for annoyance and self-reported sleep disturbance in Hong Kong are different from relationships synthesized from earlier studies - despite the western bias and temperate-climate bias in the studies available in the syntheses. This is an important finding for urban planning and traffic noise management of the growing mega-cities in the world whose built forms can be expected to reflect that of Hong Kong more than of cities in the west.

## Background

### Exposure and exposure-response to road traffic noise

The World Health Organization has highlighted urbanization, economic development and growth in motorized transport as drivers of the growing extent and intensity of environmental risk from road traffic noise [1]. Development of policy and management responses to this risk requires, inter alia, knowledge of the prevalence of exposure to road traffic noise and the relationship between exposure and its human effects.

Hammer et al. [2] note that much of the data on the prevalence of noise exposures in the United States is out-dated and inadequate. Various estimates of population exposures to environmental noise have been obtained by a range of measurement and modelling techniques [3-6]. In Europe, the Environmental Noise Directive [7] has driven more recent estimations of exposure using extensive programs of noise mapping of road traffic and other noise sources for European urban agglomerations [8-10]. The European estimates utilize harmonized noise indicators L_den_ and L_night_: the L_den_ (day-evening-night equivalent level) is a metric related to annoyance; L_night_ (night equivalent level) is a metric related to sleep disturbance. Sleep disturbance and annoyance, mostly from road traffic noise exposure, comprise the main burden of disease from environmental noise in Europe [1].

Exposure-response relationships for annoyance with road traffic noise have been estimated over many decades: amongst the earliest being in France [11], Sweden [12], the UK [13] and the USA [14]. There has been considerable variation in the results of individual studies and various syntheses have been performed [15-17]. The most recent meta-analysis was that by Miedema and Oudshoorn [18] who examined twenty-six studies from six European countries and Canada, consisting of a total of 19,172 individuals. They reported the percentage of the community Highly Annoyed (%HA) over an L_den_ exposure range of 45–75 dB together with confidence intervals for the population mean %HA. Much of the base data for this meta-analysis is now several decades old, and similarly for the revised international standard ISO 1996–1 [19] and the American standard ANSI 12.9 – Part 4 [20] which provide other yardsticks for exposure-response relationships for transport noise sources. More recent exposure-annoyance studies for road traffic noise have been reported from Europe [21,22] and from Asia [23-25] but there have been no further syntheses, and invariably the authors of any new study have benchmarked their result with the relationship reported by Miedema and Oudshoorn [18].

The effects of noise exposure on sleep have both acute and long-term dimensions, and these are associated with different noise indicators. Acute effects link with event-related measures while overall sleep parameters link with L_night_, as a whole-of-night indicator. A meta-analysis of 13 subjective self-reported sleep disturbance studies from road traffic noise (9,603 individuals from: 8 studies from Europe, 2 from Canada, 2 from Japan and 1 from Turkey) was reported by Miedema and Vos [26]. It related the percentage of the community who self-reported being Highly Sleep Disturbed (%HSD) to L_night_ over a range of 45 to 65 dB. We note that self-reported sleep disturbance is a subjective measure of the effects of noise on sleep often used in surveys, while more objective polysomnographic measures can be used in experimental settings, but are less suitable in large-scale community surveys.

### Road traffic noise and different city form

**Figure 1:**
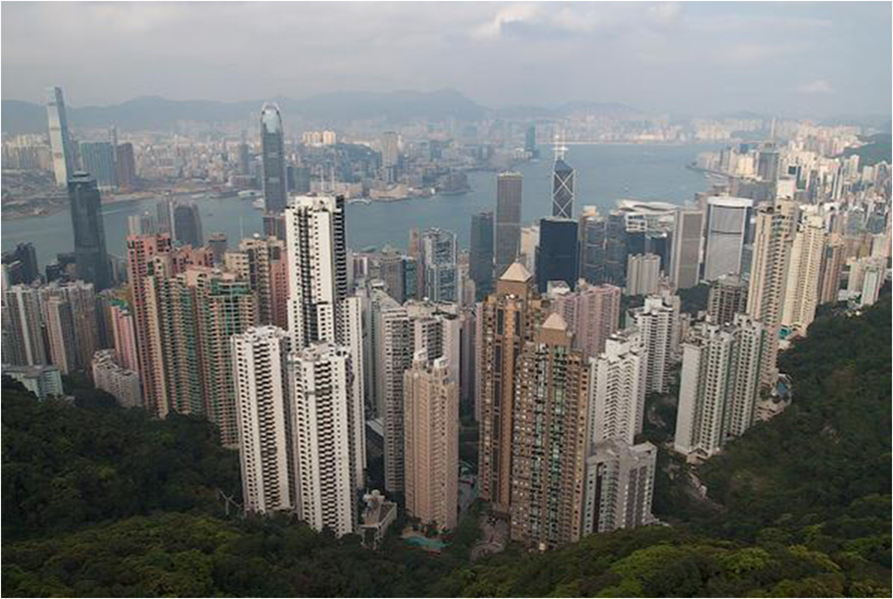
**The Hong Kong city form.** This is characterized by high population density, high-rise residential development with air conditioned apartments, and high road traffic intensity. (Photograph: VascoPlanet™).

There is a global shift in the centre of gravity of urbanization from the developed to the developing world. In the latter, about half of the population already live in cities and this proportion will be two-thirds by 2050 [27]. By 2025, more than half of the twenty-five megacities in the world will be in Asia, and located in the tropics or sub-tropics [28]. Hong Kong (Figure 1) has one of the world’s highest population densities with most of the population living in high-rise buildings, including what Yuen and Yeh [29] call super-tall buildings of 50 storeys or more, surrounded by high intensities of road traffic (251 vehicles/road kilometre [30]). Most of the dwellings in Hong Kong are apartments in these high-rise building, typically with two to three bedrooms and mostly in line of sight with nearby or distant roadways. While the city form of any individual city will depend on topography, planning controls and land economics, the growing number of large and mega-cities of Asia and elsewhere will likely be closer in morphology to that of Hong Kong than they will be to the cities of Europe and North America. The high-density vertical development and dense road traffic on a limited road network of Hong Kong are being emulated elsewhere [31].

For this reason, these results from Hong Kong have relevance well beyond this one city. An important question is whether current knowledge on exposure, and syntheses of exposure-response relationships, largely based on studies in “western” cities, are applicable in cities across the world, or whether they are shaped by the physical [32] and social characteristics of the cities in which they were conducted. Architectural forms of Hong Kong residential development are highly varied [33] but their common feature is verticality. Hong Kong’s differences also extend beyond built form: it has a monsoonal humid subtropical climate with near universal air-conditioning of domestic premises, and this will change the indoor experience of outdoor noise. Some literature also suggests that the social and cultural background of a population may affect perception of factors such as overcrowding and noise [34], and Ko [35] reported a different sensitivity of Chinese to aircraft noise. Brown [36] however, has refuted evidence of this difference.

This paper examines (1) if the exposure to road traffic noise in a high-rise city, with high population and traffic densities, is different from that in European cities; and (2) if the exposure-response relationships for annoyance and self-reported sleep disturbance in Hong Kong can be considered as drawn from the same population of exposure-response relationships as that on which the Miedema and Oudshoorn [18] and the Miedema and Vos [26] meta-analyses of annoyance and self-reported sleep disturbance responses, respectively, were based.

## Methods

A city-wide study was commissioned in the Hong Kong Special Administrative Region (HKSAR). It utilized a large random sample of the population, estimated the exposure of the dwellings of this sample to road traffic noise, and measured annoyance and self-reported sleep responses by questionnaire.

Sample selection and household interviews were conducted by the Census and Statistics Department (CSD) ([37]: Appendix one). The population sampled was all residential addresses in the HKSAR—2,292,707 households in which 6,888,080 people resided in 2010. Dwellings were randomly sampled, with prior letters sent to the selected addresses, and house calls between November 2009 and March 2010. An adult aged 18 years or above was randomly selected for interview in each selected household. The survey responses were from individuals, not aggregated over the household. A total of 10,077 interviews were successfully completed, achieving a 76% response rate overall.

The representativeness of this sample can be confirmed, in part, by comparing selected demographic and housing characteristics with those available for the whole Hong Kong population in the HKSAR 2011 census [38]. The percentages for the sample and the population in Table 1 demonstrate that the respondents in the survey provide good representation of the Hong Kong population, though there has been minor oversampling of those aged 65 years and above (likely explained by the higher probability this age group would be at home when surveyors called) and some undersampling of the two younger age categories, particularly those aged 18–24. The housing type of those in the sample reflects those in the population across public rental housing, subsidized sale flats, private housing and temporary housing.

**Table 1:** **Proportion of the sample, and the proportion of the Hong Kong population, female or male, by age category, and by housing type**

**Variable**		**% of the sample (n = 10,077)**	**% of the Hong Kong population (from 2011 Census)**
**Sex**	female (18 years and over)	53.2%	53.3%
	male (18 years and over)	46.8%	46.7%
**Age**	65 or above	19.0%	15.4%
	55-64	15.0%	15.1%
	45-54	23.1%	21.1%
	35-44	22.1%	18.6%
	25-34	14.3%	17.8%
	18-24	6.6%	12.0%
**Type of housing**	public rental housing	35.8%	30.5%
	subsidized sale flat	16.2%	15.9%
	private housing	46.3%	50.2%
	other permanent housing	1.1%	2.3%
	temporary housing	0.5%	1.1%

**Figure 2:**
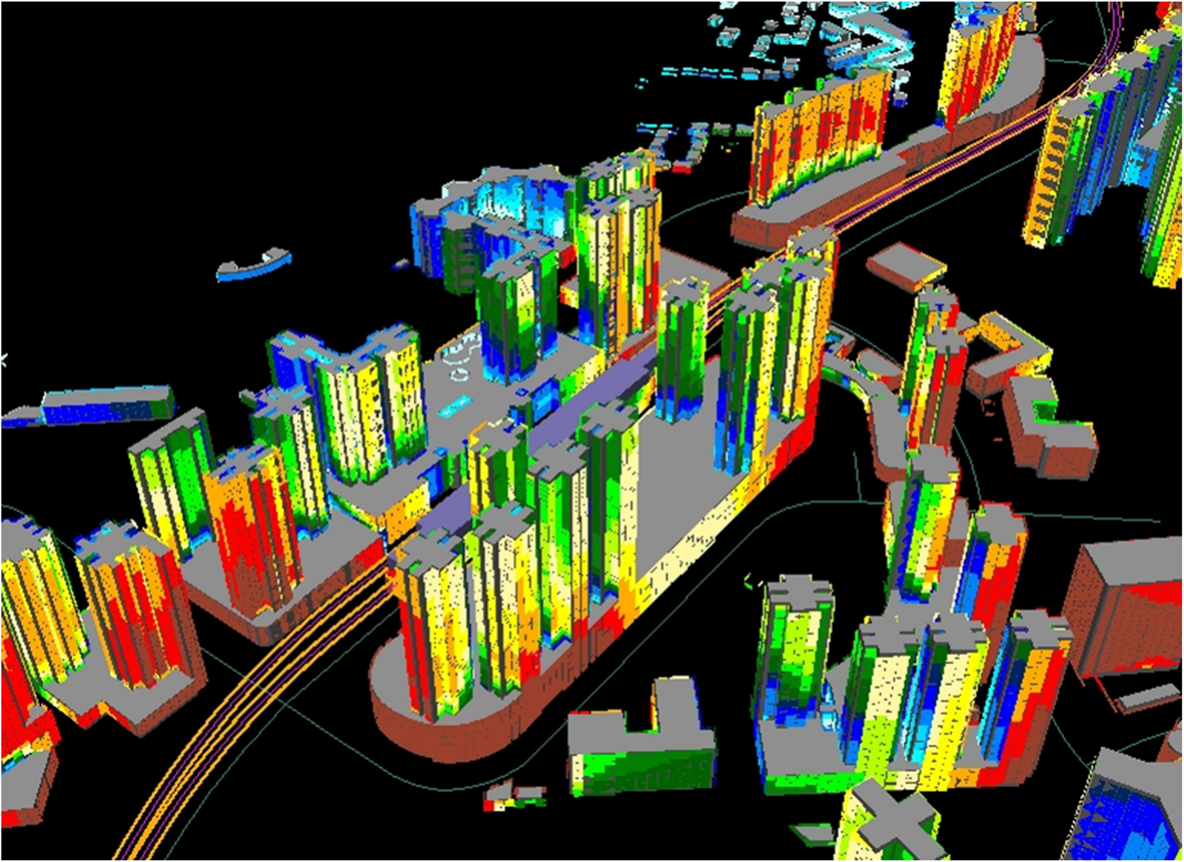
**An example of the application of 3D noise mapping of high-rise residential building facades.**

City-wide traffic noise mapping had been conducted using 3D technology [39] and the ISO 9613–2 [40] method including the LimA software [41] adapted for Hong Kong, together with digital topographic, building and traffic data for the year 2010. Figure 2 illustrates the mapping by which L_den_ and L_night_ were calculated at the most exposed façade of each of the 10,077 dwellings in the complex vertical urban form of Hong Kong.

Response to noise was measured as one component of a routine CSD Thematic Household Survey [37]. The noise focus was initially masked with the questionnaire posed as a survey on general environmental issues of the neighbourhood. Face-to-face interviews were conducted in respondents’ households. A rigorous data verification protocol applied by the CSD ensured quality control, with 15% of the households revisited to ascertain reliability of answers concerning factual matters, such as the number of rooms in the dwelling. Questionnaire design and application protocol followed the international standard for measurement of annoyance [42].

Annoyance was measured on a 0–10 numeric scale (‘Thinking about the last 12 months or so, when you are here at home, what number from 0 to 10 best shows how much you are bothered, disturbed or annoyed by road traffic?’) with’ not at all’ and ‘extremely’ as end-labels of the scale. Self-reported sleep disturbance was measured on a similar scale (‘… what number from 0 to 10 best shows how much was your sleep disturbed by noise from road traffic?’). Spoken languages in Hong Kong include Cantonese, English and Mandarin, and the questionnaire was prepared in all three. Initially in English, it was translated to colloquial Cantonese and Mandarin then translated back to English as a crosscheck. The verbal annoyance descriptors in Cantonese were derived from a pilot test akin to the procedure undertaken by Ma et al. [43] for Mandarin.

The questionnaire also included a noise sensitivity scale, using a revised version of the Weinstein scale [44] and a measure of respondent’s overall satisfaction/dissatisfaction with their residential area (defined as their residential “estate/street block”) as a place to live. The latter question was asked early in the interview before respondents were aware the survey focussed on noise.

## Results

### Exposure of the Hong Kong population to road traffic noise

**Figure 3:**
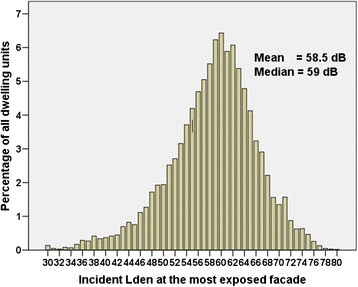
**Distribution of the exposure to road traffic noise of all dwelling units in the sample (n = 10,077).** These are predicted by 3D noise mapping. The estimates are of L_den_ in 1 dB bins for levels above 30 dB.

The level of road traffic noise (L_den_) at the most exposed façade of the 10,077 dwellings in the sample of Hong Kong households is shown in Figure 3. To facilitate comparison with exposures of European cities, these are reported as incident levels with no inclusion of the sound reflected from the building façade of interest [7,45,46] though reflections from other building facades in the area had been included in the prediction modelling.

Given the large sample size, and the random selection from a sampling frame of all households in the HKSAR, Figure 3 is a good estimate of the road traffic noise exposure of the dwellings of the Hong Kong population. L_den_ levels ranged from 30 dB to 80 dB, with a median exposure of 59 dB. This is some 4 dB higher than the estimated median of 55 dB in European cities [10] and, at least initially, appears to support suggestions that traffic noise levels may be higher in Asian than Western cities [25,47,48].

### Extent of annoyance and self-reported sleep disturbance from road traffic noise

The extent of annoyance and self-reported sleep disturbance from road traffic noise in the Hong Kong population has been estimated from the survey results. Based on responses from the annoyance, annoyance at night and sleep disturbance questions, and utilizing the same cut-offs used by Miedema and Oudshoorn [18] for 11-point scales, results are reported in Table 2 as %HA (percentage highly annoyed), %A (percentage annoyed), and %LA (percentage (at least) a little annoyed), with equivalents for self-reported sleep disturbance. The 95% confidence intervals for estimates of the proportion of the adult population in Hong Kong annoyed or sleep disturbed are also shown.

**Table 2:** **Estimates of the proportion of the population of Hong Kong annoyed, or self-reporting sleep disturbance, by road traffic noise**

	**% of the sample**	**95% confidence interval of the % in the HK population**
**Annoyed with road traffic noise (over whole day)**		
Highly annoyed	7.9%	7.4 to 8.4%
Annoyed	24.6%	23.7 to 25.4%
(at least) A little annoyed	47.8%	46.8 to 48.8%
**Annoyed with road traffic noise (at night)**		
Highly annoyed at night	5.0%	4.5 to 5.4%
Annoyed at night	15.7%	15.0 to 16.4%
(at least) A little annoyed at night	36.1%	35.2 to 37.0%
**(Self-reported) Sleep disturbed by road traffic noise**		
Highly sleep disturbed	4.1%	3.7 to 4.5%
Sleep disturbed	11.3%	10.7 to 11.9%
(at least) A little sleep disturbed	27.3%	26.4 to 28.2%

### Exposure-response relationships for annoyance and self-reported sleep disturbance in Hong Kong

**Figure 4:**
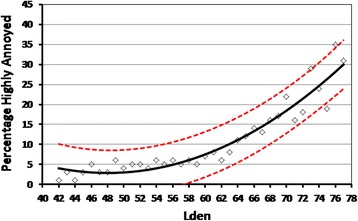
**The %HA with road traffic noise.** The data points are the %HA within each 1 dB interval of exposure over the L_den_ range of 42 to 77 dB. The best fit quadratic exposure-response regression model is shown, together with 95% upper and lower confidence bounds.

The %HA with road traffic noise was calculated within each 1 dB exposure band. Various models regressing %HA with L_den_ were examined, and a quadratic model was the best fit (R^2^ = 0.912). The fitted model over the range from 42 dB to 77 dB L_den_ is shown in Figure 4 (%HA = 77.36 – 3.102L_den_ + 0.0323L_den_^2^). Levels below 42 dB were excluded because of estimation method uncertainty at these low levels, and there were fewer than 10 respondents exposed in each 1 dB band above 77 dB and these too were excluded.

**Figure 5:**
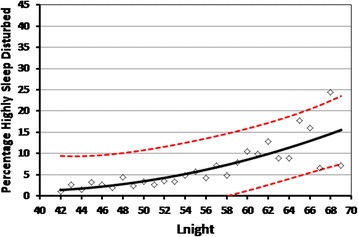
**The %HSD by road traffic noise.** The data points are the %HSD within each 1 dB interval of exposure over the L_night_ range of 42 to 69 dB. The best fit quadratic exposure-response regression model is shown, together with 95% upper and lower confidence bounds.

As for annoyance, the %HSD with road traffic noise was calculated within each 1 dB band of exposure and regressed against L_night_. Figure 5 shows the best-fit quadratic model (%HSD = 22.64 – 1.1245L_night_ + 0.0148L_night_^2^) over the range from 42 dB to 69 dB (R^2^ = 0.629). Exposures above 69 dB L_night_ were excluded because there were 10 or less respondents exposed in the 1 dB bands above this level.

### Moderating variables

Various authors e.g. [49,50] have examined confounders and effect modifiers in exposure-response relationships. In the present study, a binary logistic regression analysis (IBM SPSS Statistics, Version 21) was conducted using the exposure (L_den_) and individual annoyance outcome of all respondents in the sample (n = 10,077), to assess if select variables intervened in the relationship. For this analysis, a respondent’s annoyance was coded as “1” Highly Annoyed, for a score of 8, 9 or 10 on the 0–10 numeric annoyance scale, or “0” Not Highly Annoyed, for all other scores. Four independent variables were examined: two related to the high-rise built form of Hong Kong that could potentially be confounders, and two that were personal factors known to be significant effect modifiers from previous studies [49]. The variables included in the logistic regression were:

- Floor Level of the respondent’s quarters, categorized as low (0 to 15th floor), mid (16th to 35th floor) or high (36th to 71st floor). These category bounds were based on equal intervals of the logarithm of vertical propagation distance from surface roadway sources to higher floors;
- Public (0) or Private (1) ownership of the respondent’s living quarters. Such ownership status is a primary discriminant of the morphological characteristics of residential buildings in Hong Kong (see Reference 33, Table 1);
- Noise Sensitivity. Overall noise sensitivity scores were classified by tertile cut-offs as High, Medium or Low [51];
- respondents’ satisfaction with their residential area coded as “1” Dissatisfied and “0” Not Dissatisfied.

Predictor variables were entered in the logistic regression in blocks, with noise exposure L_den_ in the first step, the built-form variables in the second and personal variables in the third step. Table 3 shows results from the logistic regression. The Wald criterion demonstrated that Floor Level was not significant in predicting Highly Annoyed respondents. All other variables were significant, though the Public/Private ownership was only marginally so.

**Table 3:** **Binary logistic regression of highly annoyed respondents on noise exposure, testing significance levels of potential confounder variables (floor level and public/private status) and effect modifiers (noise sensitivity and overall satisfaction/dissatisfaction with the neighbourhood)**

**Independent predictor variables**	**B**	**S.E**	**Wald**	**df**	**Sig.**	**Exp(B) (odds ratio)**	**Cumulative Nagelkerke R Square**
*Block 1*							
**L**_**den**_ **exposure of respondent**	.079	.006	169.2	1	.000	1.083	.058
*Block 2*							
**Floor Level** (ref cat: low floors 0–15)							.059
**mid floors 16–35 category**	.117	.086	1.855	1	.173	1.124	
**high floors 36–71 category**	.128	.218	.346	1	.556	1.137	
**Public/Private ownership of quarters**	.167	.081	4.252	1	.039	1.182	
*Block 3*							
**Noise Sensitivity** (ref. cat.: Low NS)							.112
**Medium NS category**	.406	.110	13.71	1	.000	1.501	
**High NS category**	.889	.101	77.58	1	.000	2.433	
**Dissatisfied with residential area**	1.254	.106	140.1	1	.000	3.503	

The odds ratios in Table 3 show that respondents in medium and high Noise Sensitivity categories were 1.5 and 2.4 times more likely to be Highly Annoyed than were respondents in the low Noise Sensitivity category. Respondents who were dissatisfied overall with their residential area were 3.5 times more likely to be Highly Annoyed than respondents not dissatisfied with their area. Noise Sensitivity and overall satisfaction with the living environment, are effect modifiers. However, while the logistic regression, by including successive blocks of variables in the analysis has resulted in an increase in the Nagelkerke R Square statistic, the overall ability of the model to predict individual respondents who were Highly Annoyed remains low (Nagelkerke’s R Square = .112).

A binary logistic regression analysis of the relationship between road traffic noise exposure (L_night_) of respondents and their sleep disturbance outcomes produced analagous results and is not reported here.

## Discussion

This Hong Kong study is one of the largest exposure-response studies for road traffic noise ever undertaken. The size of the sample (n = 10,077) was greater than the total number of subjects utilized in the Miedema and Vos [26] 13-study self-reported sleep disturbance meta-analysis, and more than half of all the subjects utilized in the Miedema and Oudshoorn [18] 26-study annoyance meta-analysis. The large sample, rigorous random sampling of the HKSAR adult population, a response rate of 76%, estimation of road traffic noise exposure incident at each individual dwelling, and application of best international practice in the measurement of annoyance and self-reported sleep disturbance, have produced benchmark estimates in this study of the exposure and responses of the Hong Kong population to road traffic noise.

### Comparing exposure to road traffic noise in Hong Kong and elsewhere

**Figure 6:**
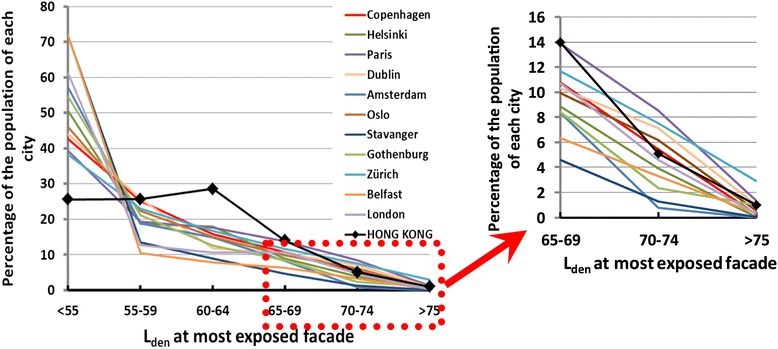
**The percentage of each city population exposed to road traffic noise (L**_**den**_ **) in 5 dB bands.** For Hong Kong and a selection of European cities [10].

The exposure of the Hong Kong population to road traffic noise can be compared with recent estimates from Europe. Figure 6 plots the Hong Kong exposure with that of a selection of European cities [10] including two with population sizes similar to Hong Kong (Paris and London), several cities of one million population, and two smaller cities. The European data is in 5 dB bands with no reporting of exposures less than 55 dB. In Figure 6, the “<55 dB” category has been calculated as the balance of the city populations whose exposure has not been estimated. The Hong Kong data, originally in 1 dB bands and estimated to levels as low as 30 dB, have been transformed into 5 dB bands in Figures 6, and concatenated to the range reported from Europe.

The differences between the Hong Kong exposures and that of the European cities are striking except at the high end of exposure, and consistent across all the comparison cities. The proportion of the population in Hong Kong exposed to the higher levels of road traffic noise is similar to that in European cities, but a much higher proportion of the Hong Kong population is exposed to levels of road traffic noise in the band 60–64 dB, and a much lower proportion to the lower levels of exposure experienced in European cities (<55 dB).

We explain this in terms of the compact and vertical nature of urban development in Hong Kong. There is close proximity of some dwellings to roadways with consequent high noise exposures, but this occurs in nearly all cities: whether in Hong Kong, Europe or North America. What is different is that, in most other cities, a significant proportion of dwellings are also located at considerable distances from major roadways (see, for example, Brown and Lam [52]) and this proportion, if the development is not high-rise, benefits from acoustic shielding provided by intermediate buildings along the propagation path—resulting in something of the order of half of the populations of many cities being exposed to lower levels of road traffic noise (say < 55 dB). The high-rise buildings together with high traffic density, in Hong Kong, result in nearly all dwellings having line-of-sight to a roadway noise source, though exposure levels at many will be moderated because of the large path length distances from the roadways to the upper floors of high buildings. This deprives them of much of the shielding effect along the source-to-receiver path that is provided by the urban fabric of low-rise cities. Thus, Hong Kong is noisier, but not predominantly in terms of intensity and extensity of higher noise levels, but through the bulge in exposures to L_den_ levels in the 55 to 69 dB bands.

**Figure 7:**
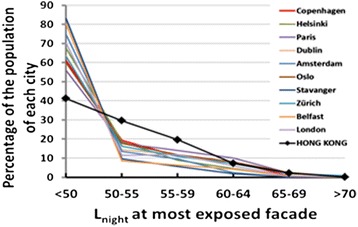
**The percentage of each city population exposed to road traffic noise (L**_**night**_ **) in 5 dB bands.** For Hong Kong and a selection of European cities of different population size.

Figure 7 makes the same comparison between Hong Kong and European cities, but in terms of road traffic noise exposures in the night hours (L_night_). The patterns of difference, and the explanations, parallel those for L_den_ in Figure 6.

### Comparing road traffic exposure-response relationships in Hong Kong and elsewhere

**Figure 8:**
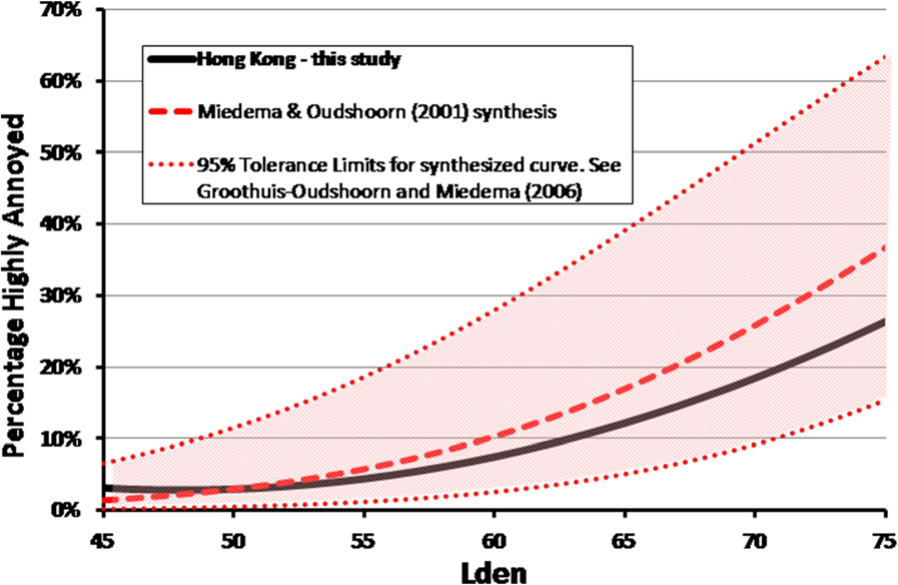
**Comparison of the exposure-response model of %HA with road traffic noise in Hong Kong with that synthesized by Miedema and Oudshoorn** [18]**.** The 95% *tolerance interval* of the synthesized exposure-response curve from the meta-analysis of 26 previous studies [18] is shown shaded.

How does the exposure-annoyance relationship for road traffic noise in Hong Kong compare to that from the Miedema and Oudshoorn [18] synthesis? Both curves are shown in Figure 8 over the range 45 to 75 dB L_den_. At levels to about 60 dB, the Hong Kong response is close to that of the population mean responses reported by Miedema and Oudshoorn [18] but at higher levels the curves diverge, with a lower %HA in Hong Kong. The differences are not particularly large: 4% and 7% at levels of 65 and 70 dB respectively, increasing to near 10% at 75 dB. The difference can also be compared by noting that, in Hong Kong, the level at which 20% of the population is Highly Annoyed with road traffic noise is 4 dB higher than estimated from the synthesized curve. The most important question is: can the relationship found in the Hong Kong study be considered as drawn from the same population of exposure-response relationships as were the original studies included in the meta-analysis?

Groothuis-Oudshoorn and Miedema [53] indicate that the tolerance interval of the exposure-response curve synthesized from their meta-analysis, rather than its confidence interval, provides bounds within which (say 95% of) any new randomly drawn exposure-annoyance curve should fall. The 95% tolerance interval for the Miedema and Oudshoorn [18] synthesis is shown shaded in Figure 8 and the Hong Kong curve falls well within this interval. Effectively, this means that, despite the western bias in the selection of exposure-response studies used in their meta-analysis, the new Hong Kong curve is from the same population of exposure-response relationships used to generate the synthesized Miedema and Oudshoorn [18] curve.

**Figure 9:**
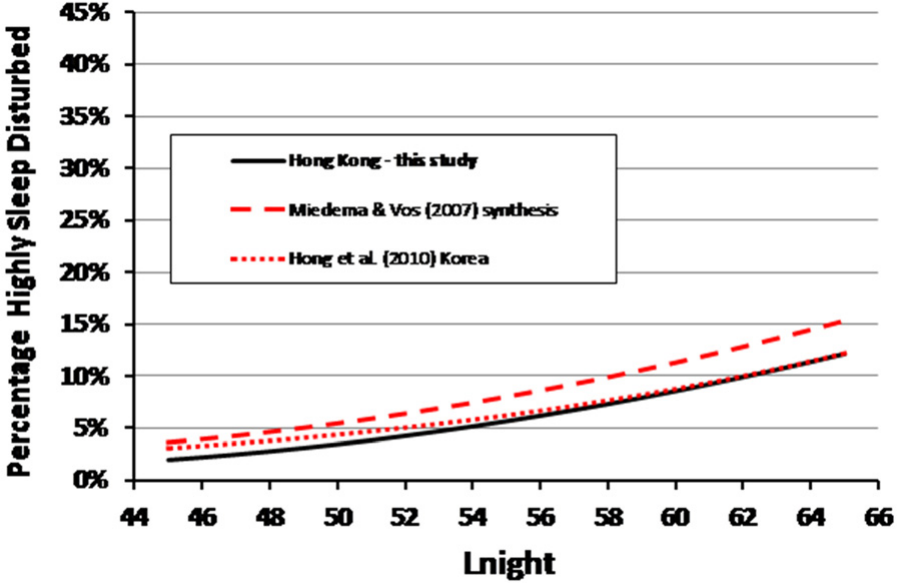
**The exposure-response model for the %HSD in Hong Kong shown with other results.** The %HSD in Hong Kong is plotted with the synthesized relationship from the Miedema and Vos [26] meta-analysis, and another single-city study from Korea [49], over the L_night_ range of 45 to 65 dB available in the meta-analysis.

The Hong Kong exposure-response curve for self-reported sleep disturbance from road traffic noise can also be compared (Figure 9) though this is only partial in the absence of tolerance limits from the sleep meta-analysis. The figure shows that the %HSD for the Hong Kong population parallels that of the Miedema and Vos [26] synthesis of previous studies, but is slightly lower. The %HSD exposure-response from a Korean road traffic study [54] is also shown. The similarity of the three curves, one based on the synthesis of largely European studies, and two from single-city Asian studies, suggests that there may not be any underlying differences between self-reported sleep disturbance from road traffic noise responses in Europe and in Asia. The Korean authors had also noted the predominance of studies of European origin included in the Miedema and Vos [26] meta-analysis.

### Other observations

While we have demonstrated that the exposure-response relationship for road traffic noise annoyance in Hong Kong falls within the tolerance limits of the synthesized curve from Miedema and Oudshoorn [18], it is still useful to consider two study-specific physical factors that could have contributed to the different, somewhat lower, mean response in Hong Kong.

The first is the potential for greater differences between external noise levels and internal noise levels as a result of the extensive use of air-conditioning in Hong Kong’s subtropical climate. Exposure-response curves are constructed on road traffic noise levels incident on the external façade, but there is logic in considering that the response may be shaped by the levels experienced inside the dwelling. Hong Kong has near universal installation of air-conditioning in dwellings—only 4% of the survey respondents reported their dwelling had none—and some 90% had air-conditioners fitted in their bedrooms, 93% in the living rooms. It is not that the acoustic properties of the window/façade material in air-conditioned premises would consistently be different to those of dwellings in other climatic zones, but the behaviour of residents with respect to ventilation may be. There is a lack of empirical data, but anecdotally the operation of air conditioning tends to be associated with complete closure of windows whereas, with the heating of dwellings in temperate climates, a high proportion of the community is known to crack windows slightly open for ventilation during sleep [55]. Complete window closure would result in lower internal noise for a given external noise exposure, potentially shifting an annoyance-response curve downwards. Future studies in both tropical and temperate climates need to measure, diurnally and seasonally, detailed window-closing behaviour.

Secondly, for dwellings with high noise exposures located many storeys above ground level, there may be a difference in the nature of the road traffic noise signal experienced. These elevations (for example say, at 60 storeys) mean long propagation paths from the surface traffic sources to the dwellings. Transmission of road traffic noise signals over these distances changes the nature of the traffic noise signal towards one with a lower variability in levels. This means that respondents in Hong Kong at higher storeys may experience a reduced “noise climate”, with maximum levels from traffic emerging less above the background traffic levels than would respondents experiencing the same L_den_ at lower floors, or as would tend to be experienced in a low-rise city. Noise events may thus be less noticeable in this situation, and there are indications that sleep disturbance from road traffic noise, and perhaps annoyance [56], may depend on the number of noise events experienced. While Floor Level of the respondent’s apartment was not a significant variable in the logistic regression analysis, differences in the noise climate experienced at different building elevations should be investigated in future studies of exposure-response in high-rise cities.

## Conclusions

The proportion of the population in Hong Kong exposed to high levels of road traffic noise (>70 dB) is similar to that found in cities in Europe. However, a much higher proportion of the population in Hong Kong compared to European cities is exposed to L_den_ levels of road traffic noise of 60–64 dB, and a much lower proportion to the lower levels (<55 dB). We have explained this as a consequence of the high-rise built form of Hong Kong where there is both high population and high traffic density. The exposure-annoyance response relationship for road traffic noise in Hong Kong falls well within the tolerance limits of the Miedema and Oudshoorn [18] synthesized exposure-annoyance curve for the percentage of the population highly annoyed with road traffic noise. Fit within a tolerance interval, rather than a confidence interval, is appropriate in comparing the exposure-response relationship from a single new study with the results of a prior synthesis of exposure-response relationships. The percentages of the Hong Kong population who reported they were highly sleep disturbed by road traffic noise also closely follows the exposure-response relationship for high self-reported sleep disturbance based on the pooled data used by Miedema and Vos [26]. There has been a Western bias, and a temperate-climate bias, in the studies used in prior meta-analyses of human responses to road traffic noise. However, the exposure-response relationships for annoyance and self-reported sleep disturbance reported from the high-density, high-rise, sub-tropical city of Hong Kong are not inconsistent with these. This is an important finding for future urban planning and traffic noise management of many of the projected mega-cities in the world that will be located in non-temperate climatic zones in Asia and elsewhere and whose urban forms can be expected to reflect that of Hong Kong more than of cities in the west.

## Abbreviations

dB: decibel
CSD: Census and Statistics Department
HKSAR: Hong Kong Special Administrative Region
L_den_: Day-evening-night equivalent sound level
L_night_: Night equivalent sound level
NS: Noise sensitivity
%HA: Percentage highly annoyed
%A: Percentage annoyed
%LA: Percentage (at least) a little annoyed
%HSD: Percent highly sleep disturbed
